# Differentiation of irradiation and cetuximab induced skin reactions in patients with locally advanced head and neck cancer undergoing radioimmunotherapy: the HICARE protocol (Head and neck cancer: ImmunoChemo and Radiotherapy with Erbitux) – a multicenter phase IV trial

**DOI:** 10.1186/1471-2407-13-345

**Published:** 2013-07-15

**Authors:** G Habl, K Potthoff, MF Haefner, A Abdollahi, JC Hassel, E Boller, M Indorf, J Debus

**Affiliations:** 1 Department of Radiation Oncology, University of Heidelberg Medical Center, Im Neuenheimer Feld 400, Heidelberg, 69120, Germany; 2 Molecular and Translational Radiation Oncology, Heidelberg Ion Therapy Center (HIT), University of Heidelberg Medical School and National Center for Tumor Diseases, German Cancer Research Center, Heidelberg, Germany; 3 Department of Dermatology, University of Heidelberg Medical Center, National Center for Tumor Diseases, Heidelberg, Germany; 4 iOMEDICO AG, Freiburg, Germany

## Abstract

**Background:**

In order to improve the clinical outcome of patients with locally advanced squamous cell carcinoma of the head and neck (LASCCHN) not being capable to receive platinum-based chemoradiation, radiotherapy can be intensified by addition of cetuximab, a monoclonal antibody that blocks the epidermal growth factor receptor (EGFR). The radioimmunotherapy with cetuximab is a feasible treatment option showing a favourable toxicity profile. The most frequent side effect of radiotherapy is radiation dermatitis, the most common side effect of treatment with cetuximab is acneiform rash. Incidence and severity of these frequent, often overlapping and sometimes limiting skin reactions, however, are not well explored. A clinical and molecular differentiation between radiogenic skin reactions and skin reactions caused by cetuximab which may correlate with outcome, have never been described before.

**Methods/design:**

The HICARE study is a national, multicenter, prospective phase IV study exploring the different types of skin reactions that occur in patients with LASCCHN undergoing radioimmun(chemo)therapy with the EGFR inhibitor cetuximab. 500 patients with LASCCHN will be enrolled in 40 participating sites in Germany. Primary endpoint is the rate of radiation dermatitis NCI CTCAE grade 3 and 4 (v. 4.02). Radioimmunotherapy will be applied according to SmPC, i.e. cetuximab will be administered as loading dose and then weekly during the radiotherapy. Irradiation will be applied as intensity-modulated radiation therapy (IMRT) or 3D-dimensional radiation therapy.

**Discussion:**

The HICARE trial is expected to be one of the largest trials ever conducted in head and neck cancer patients. The goal of the HICARE trial is to differentiate skin reactions caused by radiation from those caused by the monoclonal antibody cetuximab, to evaluate the incidence and severity of these skin reactions and to correlate them with outcome parameters. Besides, the translational research program will help to identify and confirm novel peripheral blood based molecular predictors and surrogates for treatment response and resistance.

**Trial registration:**

Clinical Trial Identifier, NCT01553032 (clinicaltrials.gov)

EudraCT number: 2010-019748-38

## Background

Squamous cell carcinomas of the oropharynx, hypopharynx and larynx are the sixth leading cancer by incidence worldwide with more than 500000 new cases a year. At the time of diagnosis most patients display signs and symptoms of locally advanced disease and have lymph node metastases [1]. SCCHN is mostly caused by tobacco and alcohol consumption and/or infection with high-risk types of human papillomavirus (HPV) [2]. SCCHN often show an overexpression of epidermal growth factor receptors (EGFR) which is described to be associated with a poor prognosis [3-6]. Standard treatment for resectable tumors is surgery including reconstruction plus post-operative radiotherapy and, for those patients found at surgery to have high risk features (extracapsular extension and/or R1 resection), post-operative chemoradiation with single agent platinum [7,8]. At present, for non-resectable patients who can tolerate it, combined concomitant chemoradiation with platinum is the standard treatment [9,10]. Organ-preservation protocols with combined chemoradiation therapy and surgery for salvage are increasingly being performed. These protocols are particularly effective for young patients with a good performance status presenting with moderately-advanced laryngeal or pharyngeal SCC. For patients who are not capable to receive standard platinum-based chemoradiation due to age, generally reduced condition and/or comorbidities, e.g. heart and renal disease or cirrhosis of the liver, the treatment of choice is radioimmunotherapy with cetuximab [7,8,11,12]. Whereas results of a standard chemoradiation are based on thousands of patients, results of the combined radioimmunotherapy are only based on about 200 patients in the experimental arm of the pivotal trial [11,12]. A study comparing the standard platinum-based chemoradiation with the novel radioimmunotherapy protocol with the anti-EGFR antibody cetuximab, however, is still missing. Hence, guidelines still recommend standard radiochemotherapy with cisplatin for patients able to receive chemotherapy [9,10].

Cetuximab is a chimeric monoclonal IgG1 antibody specifically targeting the epidermal growth factor receptor (EGFR). EGFR signal cascades are involved in cell proliferation, in cell cycle regulation, in angiogenesis, cell migration and invasion and in metastases. Cetuximab binds to the EGFR in a 5 to 10 times higher affinity than endogen ligands leading to a downregulation of EGFR molecules on the cell surface. Intracellular phosphorylation of the EGFR is inhibited and consequently the down stream signalling is deficient resulting in cell cycle arrest, increased expression of pro-apoptotic enzymes and decrease in the production of matrix metalloproteinases. Effects of EGFR inhibition that have been described are a reduction of cell proliferation, an inhibition of cell division processes and tumor growth and an increase of apoptosis [13,14]. Furthermore, cetuximab treatment leads to a decrease in the production of vascular endothelial growth factor (VEGF) blocking angiogenic processes in the tumor. Cetuximab has been found to potentiate the effects of radiotherapy in experimental systems [13-15].

Combined radioimmunotherapy with cetuximab is a well accepted treatment option in patients with locally advanced squamous cell carcinoma of the head and neck (LASCCHN) if they are not able to receive surgery or combined radiochemotherapy due to stage, reduced general condition and/or comorbidities. In the pivotal, international, randomized Phase III trial of 424 patients with locally or regionally advanced squamous cell carcinoma of the oropharynx, hypopharynx or larynx with no prior therapy, the addition of cetuximab to radiation when compared to radiation alone resulted in a higher response rate, a 9.5- month improvement in median duration of locoregional control [24.4 months versus 14.9 months], a longer disease-free survival and a 9% increase of the 5 year overall survival (OS). [11]. In retrospective comparison with chemoradiation trials, immunoradiation with cetuximab showed similar control rates in terms of overall survival (OS) and local control rate (LCR) [16]. Since the radioimmunotherapy with cetuximab is a feasible treatment option that overall shows a low incidence of systemic side effects and a favourable toxicity profile, it is especially indicated for older patients and patients with comorbidities. Recently updated EHNS-ESTRO-ESMO guidelines recommend radioimmunotherapy with cetuximab with a level of evidence of II and a recommendation grade B [8].

Skin reactions are known to be the most frequent side effect of EGFR inhibitor therapy. About 90% of patients treated with cetuximab develop an acneiform rash, but only 15% of patients develop a grade 3 or 4 skin rash [17-20]. Besides, patients on cetuximab treatment might develop other skin reactions like a xerosis cutis followed by a severe pruritus, hair and nail alterations, a stomatitis and eye abnormalities. All of those skin reactions can result in non-compliance as well as in an interruption or even discontinuation of an effective antitumor treatment approach. Besides, patients often feel anxious related to the cosmetic appearance of the rash and have the feeling to be stigmatized which can lead to psychosocial side effects and can negatively affect patient quality of life [21].

The most frequently seen side effect of radiotherapy in patients with head and neck cancer is radiation dermatitis that occurs in approximately 90% of patients. If cetuximab is administered simultaneously, an overlap of radiation and cetuximab induced skin reactions is seen in the radiation field which may lead to very severe skin toxicity [22,23]. An retrospective evaluation of acute toxicity of skin and mucosa in patients with head and neck cancer receiving radiotherapy (RT) alone or in combination with radiotherapy plus chemotherapy (RCT) or with cetuximab (RIT) published by Garcia-Huttenlocher et al. showed grade 3 overall skin toxicity in 27.6% of the RIT patients compared to 0% in patients only receiving RT and compared to 7% in patients receiving RCT [22]. Cetuximab associated acneiform rash grade 3 was observed in 7% of the RIT patients. In this publication cetuximab did not lead to a higher rate of RT interruptions compared to RT and RCT. Eight weeks after RT all patients had recovered from these dermatological side effects [22]. Bonner et al. reported on 21% of patients with grade 3/4 radiation dermatitis in the RT group. In the radioimmunotherapy group, however, a significant increase to 35% of grade 3/4 radiation dermatitis was observed. Since there is a discussion about a possible correlation between the intensity of acneiform rash and the efficacy of therapy [11], Bonner et al. conducted a subgroup analysis showing an overall survival benefit in patients developing an acneiform rash grade 2–4 compared to patients with acneiform rash grade 0–1. Such patients survived for a median of 69 months, whereas patients who had no reaction or only a mild rash had a median survival of 26 months [11]. Thus, it might be possible that the acneiform rash is a predictive biomarker for immunological response and, furthermore, that it is conductive for optimal outcome. A similar pattern has already been shown for other types of cancer [24-26]. Vermorken and colleagues,^,^ however, conducted a thorough analysis of cetuximab-induced skin reactions and acne-like rash with clinical markers of activity. Although development of skin reactions and acne-like rash appeared to trend toward improved response and disease control, early development of these toxicities was not associated with improved response or overall survival [27].

Budach et al. reported on two patients who had severe radiation dermatitis while receiving radioimmunotherapy with cetuximab [23]. Severe radiation dermatitis may occur after irradiation alone, but grade 4 lesions are rarely observed. Coexisting conditions like previously received chemotherapy or radiotherapy or even intensive sun exposure in the past may predispose for aggravation of radiation and cetuximab induced skin reaktions. Liver or renal dysfunction with possible alterations of pharmacodynamics of cetuximab may also predispose patients to develop more severe radiation dermatitis [28]. In a retrospective survey in EORTC institutes, the members observed that in 49% of patients treated with cetuximab and concurrent radiotherapy developed grade 3 or 4 radiation dermatitis [29]. Hence, the incidence of these severe skin reactions was twice as high compared to that reported by Bonner et al. [11,12]. The real percentages of grade 3 and 4 radiation dermatitis during combined radioimmunotherapy with cetuximab, however, have never been evaluated. Furthermore, the differentiation between radiation dermatitis and cetuximab induced acneiform rash seems to be important for therapeutic and prognostic reasons but has never been explored in detail so far. Thus, a prospective, multicenter large-scaled study is warranted to distinguish radiation dermatitis from cetuximab induced skin reactions, to determine the real incidence of the different types of skin reactions in a large cohort of patients, and, besides, to identify and evaluate biomarkers and surrogates correlating with clinical efficacy and outcome.

The HICARE trial is a prospective, open phase IV trial exploring the prominent side effects of skin toxicities of combined radioimmun(chemo)therapy with the EGFR inhibitor cetuximab (Erbitux®) in patients with LASCCHN in great detail. The HICARE study is a national multicenter trial in Germany, 40 centers will participate.

## Methods/design

### Study design

The HICARE study is designed as an open-label, prospective, multicenter, one-armed phase IV study according to the German drug law evaluating the incidence and severity of radiation dermatitis and acneiform rash in patients with locally advanced head and neck cancer treated with combined radioimmuno(chemo)therapy with cetuximab.

### Study objectives

Primary endpoint of the study is the rate of radiation dermatitis grade 3 and 4 according to the National Cancer Institute, Common Terminology Criteria for Adverse Events (NCI CTCAE), version 4.02. Secondary endpoints are skin related parameters like the rate of radiation dermatitis grade 1 and 2, the rate of cetuximab-induced acneiform rash grade 1–4, the rate of cetuximab-induced rhagades (all grades), the rate of cetuximab-mediated nail changes (all grades). Furthermore secondary endpoints include parameters of efficacy, i.e. objective response rate (ORR), locoregional control rate (LCR), progression free survival (PFS), overall survival (OS), safety parameters, i.e. the median dose density of radiation and the safety profile of the applied radiation protocol as well as quality of life (QoL). In addition, feasibility aspects of the treatment schedule in common routine practice and applied to patients presenting an increased comorbidity rate compared to the study population studied in the pivotal phase III trial will be evaluated. For translational research genomic analyses of peripheral blood samples are carried out at several time points during the trial. The results of the translational research will be correlated with safety parameters, clinical outcome and quality of life.

### Trial organization

The HICARE study was designed by the study initiators at the Department of Radiation Oncology of the University of Heidelberg. It is an investigator initiated trial (IIT). The trial is conducted as multicenter trial and 40 centers all over Germany will participate. The trial medication cetuximab is commercially available and it will be administrated in combination with radiotherapy as recommended by the SmPC [30}.

### Coordination

The overall coordination is performed by the Department of Radiation Oncology of the University of Heidelberg. The Department of Radiation Oncology of the University of Heidelberg is responsible for overall trial management, trial registration, database management, quality assurance and reporting. Biometrical data analyses are conducted by the CRO iOMEDICO.

### Investigators

The investigators are experienced oncologists, i.e. radiooncologists and medical oncologists, specialized in treating patients with locally advanced head and neck cancer. Patients will be recruited and treated by the investigators of the 40 participating sites in Germany (see acknowledgement).

### Quality assurance

According to the guidelines of Good Clinical Practice (GCP) and other applicable guidelines and regulations the site monitoring will be performed by an independent monitor.

### Ethics, informed consent and safety

The final protocol was approved by the leading ethics committee of the Medical Faculty of the University of Heidelberg (AFmu-387/2010), the local ethics committee of every participating center and the Paul-Ehrlich-Institute. The HICARE trial is registered at the European Clinical Trials Database (EudraCT-No. 2010-019748-38) and at http://www.clinicaltrials.gov, number NCT01553032. The HICARE study complies with the Declaration of Helsinki in its recent German version, the Medical Association’s professional code of conduct, the principles of Good Clinical Practice (GCP) guidelines and the Federal Data Protection Act. The trial will also be carried out adhering to local legal and regulatory requirements.

For each patient recruited into the study, written informed consent is essential prior to inclusion into the study after extensive information about the intent of the study, the study regimen, potential associated risks and side effects as well as potential alternative therapies. The investigator will not undertake any diagnostic measures specifically required for the clinical trial until valid consent has been obtained.

### Patient selection

A total of 500 patients with locally advanced (UICC stage III, IVA or IVB), non-metastatic, squamous cell carcinoma located in the oral cavity, oropharynx, hypopharynx, or larynx with measurable disease will be included. For participation in the trial lymph node involvement is allowed, but a spread of the tumor to other parts of the body is an exclusion criterion. Each patient will receive the combined radioimmunotherapy as described above, i.e. radiation therapy (IMRT or 3D-radiation) and cetuximab.

Inclusion criteria include:

•Signed written informed consent

•Age ≥ 18 years of age

•Eastern Cooperative Oncology Group performance status (ECOG) of 0 to 2

•Life expectancy > 6 months

•Histologically confirmed locally advanced (stage III or IVA, IVB), non-metastatic squamous cell carcinoma of the oral cavity, oropharynx, hypopharynx and larynx (T_2-4_, N_x_, M_0_).

•Adequate bone marrow, liver and renal function according to SmPC of cetuximab based on laboratory assessments raised within 7 days prior to start of study treatment.

•Women of childbearing potential must have had a negative serum or urine beta-HCG pregnancy test within 7 days prior to the first administration of study treatment or must have a documented condition that prohibits pregnancy (e.g. post-menopausal; hysterectomy).

•Commitment to use of adequate contraception: patients enrolled in this trial must be willing to use effective birth control measures during the course of the trial and the subsequent 2 months

Exclusion criteria include:

•Previous radiotherapy for carcinoma of the head and neck

•Nasopharyngeal carcinoma

•Distant metastases

•Prior exposure to EGFR pathway targeting therapy

•Surgery within the last 30 days

•Known allergic/hypersensitivity reaction to any drug scheduled for the study treatment

•Previous or concurrent cancer within 5 years prior to study entry that is distinct in primary site or histology except adequately treated basal cell carcinoma or preinvasive cervical carcinoma.

•Substance abuse, medical, psychological or social conditions that may interfere with the patient’s participation in the study or evaluation of the study results as judged by the investigator

•Any condition that is unstable or could jeopardise the safety of the patient and their compliance in the study as judged by the investigator

•Legal incapacity or limited legal capacity

•Pregnant or breast-feeding women

### Work-up

Routine staging includes physical examination, chest X-ray, head and neck panendoscopy under anaesthesia, and head and neck computed tomography (CT) scan or magnetic resonance imaging (MRI) and abdominal ultrasound. All patients will be evaluated for head and neck surgery. In case resection is either surgically or medically impossible or the patient refuses to undergo the procedure, standard chemoradiation with carboplatin and 5-FU (without cetuximab) will be chosen according to current guidelines. In case that primary chemoradiation is medically impossible due to comorbidities, reduced general condition, in- and exclusion criteria for radioimmunotherapy will be evaluated and patients will be checked for eligibility for the HICARE trial. It is to note that a combination of the applied radioimmunotherapy with a chemotherapy in terms of a combined radioimmunochemotherapy (e.g. with cisplatin or carboplatin/5-FU) is not an exclusion criteria and, if applied, will be evaluated separately. Patients previously treated with induction chemotherapy are allowed to participate in this trial. Should a patient be eligible, information about participation in the study including potential risks and benefits is given to the patient. As soon as written consent is obtained, patients can be included into the trial and the required documentation will be provided by the study center (Clinical Trial Center, Department of Radiooncology, University of Heidelberg Medical Center).

After inclusion, for planning of the radiotherapy each patient receives a CT-scan in an individually adjusted precision immobilization device.

If a patient refuses treatment within the HICARE trial, the same standard combined radioimmunotherapy will be offered if chemoradiation is not applicable.

### Investigational drug according to German drug law

#### Cetuximab (Erbitux®)

The commercial available monoclonal antibody cetuximab will be applied according to the German SmPC [30]. Cetuximab is given with a loading dose of 400 mg/m^2^ of body surface as an intravenous infusion on day 1. Subsequently, the regular weekly dose during the radiotherapy is 250 mg/m^2^ of body surface on days 8, 15, 22, 29, 36 and 43. A prophylactic premedication with corticosteroids and antihistamines is required to reduce the incidence of infusion-related reactions such as allergic or hypersensitivity reactions.

#### Radiation therapy

Irradiation is applied as modern standard radiotherapy for LASCCHN, i.e. intensity modulated radiation therapy (IMRT) or 3D-conformalradiotherapy. Within the HICARE trial, the standard radiotherapy scheme of the institution can be applied. A summary of radiation protocols routinely used for this indication is given in Table 1. Radiation therapy can be carried out on an outpatient basis unless the patient’s condition requires hospital admission.

**Table 1:** Possible radiotherapy protocols for treatment of patients with LASCCHN according to Bonner et al. (12), plus modification for IMRT

**Regime**	**Total dose**	**Single dose (once a day)**	**Single dose (twice a day)**
Once a day	70 Gy in 35 fractions	2 Gy/fraction; 5fractions/weekduration: 7 weeks	--
Twice a day	72-76,8 Gy in 60–64 fractions	--	1,2 Gy/fraction; 10 fractions/week Duration: 6–6,5 weeks
Concomitant boost	72 Gy in 42 fractions	32,4 Gy; 1,8 Gy/fraction; 5 fractions/week duration: 3,6 weeks	a.m.: 21,6 Gy; 1,8 Gy/fraction; 5 fractions/week Duration: 2,4 weeks p.m.: 18 Gy; 1,5 Gy/fraction; 5 fractions/week Duration: 2,4 weeks
(helical) IMRT	66 Gy in 30 fractions	Intergrated Boost 5 fractions /week duration: 6 weeks	--

#### Supportive therapy

Antihistamines such as clemastine or dimentinden and steroids are administered intravenously prior to the application of cetuximab according to the SMPC [30]. Skin reactions, especially acneiform rash and xerosis cutis, are treated according to current treatment recommendations. Whenever it is necessary, metoclopramide or 5-HT_3_-antagonists are used for antiemetic treatment.

Radiation induced skin reactions are treated with mild moisturizing lotion according to in-house protocols.

### Translational research

#### HPV

Patients will not be selected by human papillomavirus (HPV) status, but since HPV is increasingly being found in head and neck cancers and since there is a prognostic importance of the HPV status [2,31-33], tumor samples will be tested for HPV status. It remains to be seen if the particular subgroup of patients with HPV-positive tumors is the one in the HICARE trial that may benefit more from the addition of cetuximab and may have better outcomes than those with HPV-negative tumors.

#### Blood samples

For the translational research program, genomic DNA and Total-RNA (including micro RNAs) of peripheral blood samples will be collected from each subject prior to the first administration of cetuximab (day 1), additionally during the treatment on day 8 (prior to the second administration of cetuximab) and on day 22 (prior to the fourth administration of cetuximab). The goal of these studies is to identify molecular predictors of normal tissue and tumor response to combined radioimmunotherapy using transcriptomics, microRNA or epigenomic approaches.

#### Assessment of skin reactions

Skin-related toxicities like cetuximab-induced acneiform skin rash and radiation dermatitis will be graduated by a physician in every participating center. Each week just before the cetuximab application a photo series will be taken to get an objective measure for grading skin toxicities. Photos will be taken with the same type of camera. These photos will be blinded and separately reevaluated by experienced physicians authorized by the sponsor, i.e. a radiooncologist, a medical oncologist and a dermatologist. Based on the photo documentation it will be possible to define the correct graduation of the acute skin toxicities and, besides, to compare them with historical data from the Bonner trial [11,12] if using NCI CTCAE v. 4.2 and the former version 3.0 to receive this comparability with the pivotal trial [34,35].

#### Quality of life assessment

Assessment of quality of life is one of the secondary objectives of the trial. The following questionnaires will be used: EORTC QLQ-C30, the head and neck cancer-specific module EORTC QLQ H&C as well as the Dermatology Life Quality Index (DLQI). The EORTC QLQ30 is a general measure of quality of life in cancer patients, whereas the DLQI is a specific measures 10-question validated questionnaire which is frequently been used in dermatology. These assessments will be performed at the baseline visit, on day 22 during the course of the treatment, within the last week of radiotherapy and at the first follow-up visit.

### Safety and discontinuation of treatment

Radiotherapy-related toxicities will be assessed using the NCI Common Toxicity Criteria (CTCAE v.4.2) and the outdated version 3.0 in order to achieve a comparison to former studies, furthermore by duration, onset, and relationship to study treatment [34,35]. Toxicity will be evaluated at baseline, weekly during radiation therapy (blood count, electrolytes, chemistry, clinical examination, patient visits) and at follow-up visits. Unacceptable toxicity is defined as unpredictable or irreversible grade 4 toxicity. Expectable possible acute radiation-induced toxicities (up to 3 months after irradiation) comprise skin toxicity (desquamation, epitheliolysis, edema, bleeding, necrosis, erythema, hyperpigmentation, rash, pruritus), nausea, vomiting, fatigue, weight loss, loss of appetite, pneumonitis, haematological toxicity including leukopenia, thrombocytopenia or anaemia. Adequately treated these symptoms could be resolved within two to three weeks. However, transient parenteral nutrition therapy and hydration might be necessary in some patients. All acute radiation-induced toxicities should completely resolve within a few weeks post radiation therapy.

Cetuximab-induced adverse events are treated according to SmPC and according to current treatment recommendations [21]. Importantly, according to SmPC cetuximab should be delayed for up to two subsequent infusions in patients facing a grade 3 acneiform rash. Cetuximab can be restarted after resolution of the rash to grade 2 or less. Cetuximab needs to be delayed on a second or third occurrence of a grade 3 skin reaction for up to two consecutive cycles and dose reduction to 200 mg/m^2^ or 150 mg/m^2^, respectively, will be needed. Any further occurrence of grade 3 acneiform rash will lead to discontinuation of cetuximab treatment according to the recommendations of the SmPC.

### Adverse events

Patients’ toxicities are to be classified and documented according to the NCI Common Toxicity Criteria v. 4.2. Toxicity is documented weekly during RIT and prior to each application of cetuximab as well as at the follow-up visit. The investigators will report all Serious adverse events (SAE) immediately to the sponsor or his legal representative. Serious adverse events (SAE) are any untoward medical condition that occur and result in death, are life-threatening, require hospitalization or prolongation of existing inpatient’s hospitalization, result in persisting or significant disability or incapability, or are a congenital abnormality of birth defect. For all SAE, the documents and patient data must be verified by the responsible study personnel.

### Statistical considerations

The primary objective is the rate of radiation dermatitis CTC AE grade 3 and 4 and will be calculated based on the individual events as reported by the investigators. Bonner et al. [12] reported an incidence of radiation dermatitis of 18% for patients treated with radiotherapy alone compared to 23% for patients treated with radiotherapy and cetuximab. Based on data of this pivotal trial, 500 patients (with a drop-out rate of 5%) will be needed to show the 95% confidence interval to stay within the limits of 4% uncertainty (binomial test). The sample size is also sufficient to analyse the secondary objectives. The statistical analysis will be carried out using the updated versions of Statistica (StatSoft Europe GmbH) and R (http://www.r-project.org). Descriptive analyses will be done of all documented data. To compare the study population of the HICARE trial with the data of the pivotal trial by Bonner et al. [11,12], descriptive statistical methods without formal testing will be used. The analysis of the primary objective and further safety parameters will be done with all patients who received at least 1 dose of study medication cetuximab and at least 1 fraction of RT. Analyses of efficacy parameters will be done by the intention to treat population. Details on the evaluation can be found in the statistical analysis plan prepared for the study and which will be finalized and authorized by the study biostatistician and the principal investigator prior to performing any analysis.

### Objective response evaluation

Objective response will be evaluated according to RECIST criteria v.1.01 [36].

## Discussion

For patients with LASCCHN, cetuximab plus radiotherapy significantly improves overall survival at 5 years compared with radiotherapy alone, confirming cetuximab plus radiotherapy as an important treatment option in this group of patients [11,12]. However, there are no data available about a formal comparison between the combination of radiotherapy with cisplatin or cetuximab. The current guidelines still recommend using standard radiochemotherapy in patients with LASCCHN fit enough to undergo chemotherapy because there is no randomized, prospective trial comparing radiochemotherapy versus immunoradiotherapy in this patient group [8,37]. Chemoradiation is known to be associated with significant toxicities and, furthermore, its efficacy is questioned in the elderly population. The magnitude in effect of the radioimmunotherapy with cetuximab, however, is similar or even better than that achieved by chemoradiation and it proved to be less toxic [8,11]. The control rates were the same in retrospective comparison with radiochemotherapy trials [16].

For better patient selection, i.e. for definition of a patient cohort that benefits more from the less intense and less toxic radioimmunotherapy regimen and a patient cohort that benefits more from the more intense chemoradiation-based approach, patient characteristics including clinical and molecular prognostic markers and predictive surrogates have to be defined. Several clinical characteristics of patients with HNSCC have been linked to favourable prognosis, including non-smoker, minimum exposure to alcohol, good performance status, and absence of co-morbid disorders [38]. Several studies have shown a correlation between genetic aberrations, i.e. a p53 mutation, and lower response rates to chemotherapy and shorter overall survival times [39-42]. The HPV-status is associated with outcome, too [2]. To date in LASCCHN specific genetic alterations, however, are not known [43]. Testing for genetic and proteomic alterations in a large patient cohort may define prognostic and predictive biomarkers that help to identify patients who are at great risk for progression or treatment resistance and patients who might benefit most from radioimmunotherapy.

Skin reactions, the most common side effect of radiotherapy and also of anti-EGFR-treatment strategies like cetuximab, have not been evaluated and characterized in detail. Interestingly, almost all studies addressing EGFR-inhibitor-induced skin rash done in patients with metastatic colorectal cancer suggest that its severity is a suitable surrogate marker for efficacy of anti-EGFR therapy [44-47]. In patients with LASCCHN, the pivotal trial by Bonner et al. also showed that cetuximab-treated patients with prominent cetuximab-induced acneiform rash grade 2 or above had a better overall survival than patients with no or grade 1 rash [11]. A retrospective exploration of several trials done with panitumumab, another anti-EGFR monoclonal antibody, confirmed an association between clinically graded skin toxicity and patient-reported outcome, quality of life, longer progression-free survival and overall survival [26]. Other trials in patients with metastatic colorectal cancer and other solid tumors did not show any correlation between skin rash and efficacy of anti-EGFR therapy [48].

Thus, a clinical trial is warranted in patients with LASCCHN to evaluate the efficacy and toxicity of the radioimmunotherapy with cetuximab in a large patient cohort and to focus on biomarker research to better select patients who might benefit better from this treatment approach than others.

The HICARE study will be one of the largest clinical trials ever being performed in patients with LASCCHN. It will help to better understand the different clinical characteristics of skin reactions induced by radiotherapy and by cetuximab treatment, to identify molecular predictors and surrogates for treatment response and resistance and to better define a patient population where a less toxic regimen like the radioimmunotherapy with cetuximab would constitute the primary treatment option and other patient populations where a more intense chemoradiation-based approach is needed.

## Abbreviations

CRO: Clinical research organization; EGFR: Epidermal growth factor receptor; GCP: Good clinical practice; Gy: Gray; RECIST: Response evaluation criteria in solid tumours; RIT: Radioimmunotherapy; SmPC: Summary of product characteristics.

## Competing interest

The other authors declare that they have no competing interests.

## Authors’ contributions

GH, KP and JD planned and coordinate the study. GH, KP, MH, JH, and JD are conducting the study. Medical care is covered by GH, KP and MH. GH, KP and JD are responsible for the patient recruitment. EB performed the statistical calculations and will be responsible for the final statistical analysis. MI represents iOMEDICO AG, the CRO responsible for document development, eCRF programming and general study logistics. All authors read and approved the final manuscript.

## Pre-publication history

The pre-publication history for this paper can be accessed here:

http://www.biomedcentral.com/1471-2407/13/345/prepub
